# Operation Techniques and Outcomes in Patients with Chronic Limb Threatening Ischaemia Undergoing Minor Amputations—A Cohort Study

**DOI:** 10.1016/j.ejvsvf.2026.05.001

**Published:** 2026-05-14

**Authors:** Kasper H.F. van der Heijden, Erwin J. Peters, Marie-José P.J. Vleugels, Jan Willem H.C. Daemen, Barend M.E. Mees, Willemien van de Water

**Affiliations:** a Department of Vascular Surgery, Maastricht University Medical Centre, Maastricht, The Netherlands; b Department of Orthopedics, Maastricht University Medical Centre, Maastricht, The Netherlands

**Keywords:** Chronic limb threatening ischaemia, Minor amputation, Surgical technique, Wound healing

## Abstract

**Introduction:**

For patients with chronic limb threatening ischaemia (CLTI), minor amputations are frequently performed; however, evidence regarding surgical technique and outcomes is limited. This study aimed to investigate the association between toe amputation technique and clinical outcomes.

**Method:**

A retrospective cohort study was conducted, including all patients with Rutherford 5 or 6 CLTI who underwent a toe amputation at a single university centre between January 2018 and December 2023. Data were extracted from electronic patient records. Surgical techniques included metatarsophalangeal disarticulation, metatarsophalangeal disarticulation with cartilage removal, and transmetatarsal ray decapitation. Primary outcomes were wound healing status at 30 days after the procedure and time to wound healing. Secondary outcomes included complications, length of stay, additional interventions, additional amputations, and overall mortality rate.

**Results:**

One hundred and seventy-eight patients were included, with a median age of 72.2 years. Wound healing at 30 days and median time to wound healing were not associated with amputation technique (overall healing rate 68.0%, *p* = 0.16; median healing time eighty-three days (interquartile range 31.3, 146.3 days), *p* = 0.21). In multivariable analysis, toe pressure < 30 mmHg, diabetes mellitus, and procedures performed in an acute setting were independently associated with non-healing at 30 days. Overall, 34.3% of patients underwent additional revascularisation and 30.9% required a subsequent minor or major amputation. During a median follow up of 1.6 years, 91 patients (43.1%) died.

**Conclusion:**

The toe amputation technique was not associated with wound healing outcomes in CLTI. Clinical decision making should prioritise optimisation of vascular status and patient specific factors over the choice of amputation technique alone.

## Highlights

• Techniques included exarticulation, cartilage removal, or ray decapitation.
• The technique had no major impact on healing; outcomes remained poor overall.
• Healing was slow, with high re-intervention rates and poor survival outcomes.
• These findings highlight the need for good patient selection and pre-operative assessment.

## Intoduction

Peripheral artery disease (PAD) affects over 200 million people worldwide^1^ and is associated with an increased risk of other cardiovascular diseases, including ischaemic heart disease and cerebrovascular diseases.^2^ Chronic limb threatening ischaemia (CLTI) is a severe stage of PAD, affecting approximately 5–10% of patients with PAD, often leading to tissue loss.^3^ The diagnosis is associated with a 40–60% mortality rate at four years.^4^, ^5^, ^6^, ^7^

The management of CLTI encompasses revascularisation, infection control, and pressure offloading. In patients classified as Rutherford category 5 or 6 CLTI, treatment often additionally involves advanced wound care and may necessitate minor or major amputations. Annually, 3300 lower limb amputations are performed in The Netherlands, with most of these procedures being minor amputations, i.e., below the ankle.^8^ The need for a minor amputation sets the risk of requiring a subsequent amputation at approximately 22–34%, with some studies even reporting 51.2%, and is associated with developing new ulcers.^7^^,^^9^, ^10^, ^11^ The significant risk of additional amputations is due to possible progression of vascular disease, ongoing infection, or disturbed foot architecture after amputation. In addition, the concept of dieback, defined as progressive tissue necrosis extending proximally from the initial amputation margin due to inadequate perfusion or ongoing infection, plays a crucial role in the failure of minor amputations. Dieback contributes substantially to the need for further amputations, even when the initial surgical level appears clinically viable at the time of surgery.^12^

The choice of toe amputation technique, including disarticulation (through the metatarsophalangeal [MTP; or interphalangeal joint]), disarticulation with cartilage removal, and transmetatarsal ray decapitation, is based on wound characteristics, vascular status, patient characteristics, and the surgeon’s preference and experience.^13^ Amputation technique by itself may affect not only the healing process but also the risk of re-interventions.^13^^,^^14^ However, there are no clinical data comparing toe amputation techniques in patients with CLTI.

Arguments for different techniques are based on expert opinion only. On the one hand, removing cartilage may improve and speed up the adherence of bone to soft tissue.^14^ Furthermore, exposed cartilage following disarticulation may be a source of infection.^14^, ^15^, ^16^ On the other hand, intact cartilage keeps the bone marrow cavity closed and may thereby prevent infection and bleeding while simplifying surgery.^14^^,^^17^ Transmetatarsal ray decapitation may result in improved wound healing by being a more proximal resection including more soft tissue coverage. In contrast, ray decapitation may increase the risk of transfer ulcers due to destruction of the foot arch.^18^^,^^19^

Summarising, it remains unclear whether type of toe amputation technique is associated with wound healing. This study aimed to investigate the clinical outcomes of different toe amputation techniques in patients with Rutherford 5 or 6 CLTI.

## Study design

The study was designed as a retrospective cohort study to describe the characteristics and outcomes of different toe amputation techniques for wound healing in patients with CLTI. The study population consisted of all patients with Rutherford 5 or 6 CLTI who underwent a toe amputation at a single university hospital from January 2018 to December 2023. In order to maximise patient inclusion and to have a real world representation of patients, no restrictions were applied with respect to patient age, elective *vs.* emergency setting, degree of ischaemia, disease severity, infection status, or history of revascularisation. Inclusion in the study was restricted to patients with CLTI who were deemed suitable and willing to undergo a toe amputation. The results are therefore not applicable to those who undergo auto-amputation, palliative care, or more proximal amputations.

## METHOD

All patients received peri-operative assessment of pedal perfusion. Revascularisation was performed for toe pressure < 30 mmHg, or for higher toe pressure in patients with deteriorating wound conditions. Deteriorating wound conditions were classified as progression of necrosis or infection. Pedal perfusion was evaluated within an approximately four week window surrounding the date of surgery. This time frame was chosen to adequately represent the vascular status around the time of the procedure. Where applicable, measurements after revascularisation were used. If these were unavailable, pre-revascularisation measurements were used. Pedal perfusion was assessed by toe pressure, based on photoplethysmography (MedCaT B.V., Klazienaveen, The Netherlands). When toe pressure measurements were unavailable, transcutaneous oxygen pressure (TcPO_2_) values, measured in millimetres of mercury, were used. The cutoff for TcPO_2_ was < 30 mmHg. Chronic kidney disease was defined as an estimated glomerular filtration rate < 60 mL/min/1.73 m^2^, in accordance with local clinical standards.

For infection, cultures were taken, and antibiotics were prescribed in accordance with local protocols. Synthetic bone fillers, with or without antibiotics, and local antibiotics were not used. The choice of amputation technique, toe disarticulation, toe disarticulation with cartilage removal, and transmetatarsal ray decapitation, was determined by the treating surgeon performing the amputation on the basis of the local clinical situation and personal preference. Patients underwent either a planned surgical procedure (elective) or an acute procedure after admission through the emergency department or outpatient clinic. Following surgery, the wounds were either left open or closed using transcutaneous sutures. The choice of closure technique was based on the local clinical situation and the surgeon’s preference.

Data were collected retrospectively from electronic medical records. The collected variables included demographic information and relevant medical history, pre-operative vascular status, surgical details, and outcomes. A non-“Wet medisch-wetenschappelijk onderzoek” statement was issued by the Medical Ethics Committee of the Maastricht Universitair Medisch Centrum+ , and the study was granted ethical approval (2021-2995).

### Outcomes

The primary outcome variable was wound healing status at 30 days after surgery (± 5 days), defined as healing or non-healing. The criteria for wound healing status are depicted in Supplementary Table S1, as validated by Squiers *et al.*^20^ In addition, time to complete wound healing was assessed. Time to wound healing was defined as the duration until the removal of transcutaneous stitches in healed wounds or when documented as being healed, defined as complete re-epithelialisation, both retrieved from electronic patient records. These data were collected from outpatient clinic data.

Secondary outcomes were complications, length of stay, additional interventions, additional amputations, and overall mortality rate. Complications included local and systemic complications. Additional interventions for the index limb included both endovascular and open revascularisation. Additional amputations were categorised as minor (below the ankle) or major (above the ankle).^21^ Mortality data were captured using linked data between the electronic medical files and the data of the Central Bureau of Statistics in The Netherlands. Patients were followed until 1 May 2024.

### Statistical analyses

Statistical analyses were performed using IBM SPSS Statistics version 28.0 (IBM Corp., Armonk, NY, USA). Continuous variables were compared using the independent *t* test or Mann–Whitney *U* test, depending on the distribution of the data. Data distribution was assessed using the Kolmogorov–Smirnov test. Categorical variables were analysed using Pearson’s chi square test. Logistic regression analysis was used to evaluate the univariable and multivariable association with thirty day healing status. Next to amputation technique, multivariable analysis included diabetes, toe pressure, smoking, setting, and per-operative cultures because these were deemed potential confounders. Additional sensitivity analyses were performed to assess the robustness of the results. Kaplan–Meier curves were established for time to wound healing and death.

The article was prepared according to the Strengthening the Reporting of Observational Studies in Epidemiology guidelines for observational studies.^22^

## Results

### Study population

From 1 January 2018 until 31 December 2023, 178 patients with Rutherford 5 or 6 CLTI underwent a toe amputation. The median age of all patients was 73.0 years (interquartile range (IQR) 66.3, 79.8 years), with the majority being men (74.2%). Most patients lived independently at home (60.7%). A substantial proportion of patients had diabetes (68.0%), hypertension (83.7%), and ischaemic heart disease (40.4%). Most patients received five or more medications (79.2%). Twenty-six patients underwent amputation of multiple toes during the same surgical session. Overall, 173 patients (97.2%) underwent objective peri-operative assessment of pedal perfusion. A toe pressure or TcPO_2_ < 30 mmHg was observed in 36 patients (20.2%), whereas 137 patients (77.0%) had values ≥ 30 mmHg. Unless stated otherwise, perfusion measurements used for analysis reflect the pre-operative value closest to toe surgery, including measurements obtained before any peri-operative revascularisation.

Overall, 113 patients had a revascularisation < 6 months before surgery, including six who had it simultaneously with the amputation procedure. Endovascular, open, and hybrid revascularisation procedures accounted for 77.9%, 12.4%, and 9.7% of cases, respectively. The remaining 65 patients had no revascularisation within six months before surgery. Antibiotic therapy was initiated within six weeks before surgery in 75.8% of patients. Surgical procedures were performed in an acute or semi-acute setting in 47% of cases and were planned electively in 53% of cases.

Of the 36 patients with a recorded toe pressure (TP) of < 30 mmHg, 30 patients underwent peri-operative revascularisation; seven patients had a persisting TP < 30 mmHg (failed revascularisation; seven patients improved to a TP ≥ 30 mmHg), but this was measured more than four weeks after surgery; 16 patients had no post-revascularisation measurement. Six patients with a recorded TP of < 30 mmHg were not revascularised (‘no option’ patients).

Baseline characteristics stratified by amputation technique are summarised in Table 1. Patient characteristics were similar across cohorts of amputation technique except for per-operative cultures and wound closure. Patients undergoing transmetatarsal ray decapitation more often had per-operative cultures taken (74.7%) and more frequently had their wounds left open (44.8%) compared with those in the disarticulation group (43.3% and 24.3%, respectively) and the disarticulation with cartilage removal group (33.3% and 33.3%, respectively) (*p* < 0.001 and *p* = 0.027, respectively).

**Table 1:** Baseline characteristics by amputation technique.

Patient characeteristics	MTP disarticulation (*n* = 70)	MTP disarticulation with cartilage removal (*n =* 21)	Transmetatarsal ray decapitation (*n* = 87)	*p* value†
Age at procedure – y∗	72.0 [63.5, 78.5]	74.5 [71.5, 81.5]	76.0 [68.8, 80.3]	0.19
*Sex*				0.59
Male	51 (72.9)	14 (66.7)	67 (77.0)	
Female	19 (27.1)	7 (33.3)	20 (23.0)	
*Living situation*				0.64
Independent at home	47 (67.1)	13 (61.9)	48 (55.2)	
At home with help	19 (27.1)	6 (28.6)	31 (35.6)	
Healthcare institution	4 (5.7)	2 (9.5)	8 (9.2)	
BMI∗ – kg/m^2^	27.6 [24.1, 30.6]	29.1 [24.0, 31.3]	27.1 [23.5, 30.8]	0.83
Diabetes	46 (65.7)	14 (66.7)	61 (70.1)	0.83
Ischaemic heart disease	25 (35.7)	10 (47.6)	37 (42.5)	0.53
Heart failure	15 (21.4)	2 (9.5)	15 (17.2)	0.45
Asthma or COPD	10 (14.3)	2 (9.5)	10 (11.5)	0.80
Dialysis	9 (12.9)	3 (14.3)	15 (17.2)	0.74
Hypertension	58 (82.9)	18 (85.7)	73 (83.9)	0.95
Chronic kidney disease	20 (28.6)	11 (52.4)	38 (43.7)	0.13
Polypharmacy	56 (80.0)	17 (81.0)	68 (78.2)	0.94
*Tobacco use*				0.22
Never smoked	24 (34.8)	8 (38.1)	35 (40.2)	
Ex smoker	27 (39.1)	12 (57.1)	31 (35.6)	
Active smoker	18 (26.1)	1 (4.8)	21 (24.1)	
Peri-operative toe pressure∗ – mmHg	36.5 [28.0, 103]	20.0 [14.8, 81.3]	36.5 [18.5, 76.8]	0.12
Peri-operative TcPO_2_∗ when toe pressure not applicable – mmHg	35.0 [12.3, 81.8]	34.0 [14.8, 71.3]	22.0 [8.5, 54.5]	0.52
Revascularisation <6 mo before surgery	41 (58.6)	10 (47.6)	62 (71.3)	0.071
Antibiotics <6 wk before surgery	49 (70.0)	15 (71.4)	71 (81.6)	0.21
*Setting*				0.058
Acute	27 (38.6)	8 (38.1)	49 (56.3)	
Planned	43 (61.4)	13 (61.9)	38 (43.7)	
*Per-operative cultures*				<0.001
Negative	3 (4.3)	1 (4.8)	4 (4.6)	
Positive	28 (40.0)	6 (28.6)	61 (70.1)	
Not taken	39 (55.7)	14 (66.7)	22 (25.3)	
*Wound closure*				0.027
Left open	17 (24.3)	7 (33.3)	39 (44.8)	
Transcutaneous stitches	53 (75.7)	14 (66.7)	48 (55.2)	

Values in parentheses are percentages unless indicated otherwise. BMI = body mass index; COPD = chronic obstructive pulmonary disease; MTP = metatarsophalangeal; TcPO2 = transcutaneous oxygen pressure.

∗ Values are medians [interquartile range]. Kolmogorov–Smirnov test was used to assess the variance of continuous outcomes.

† Independent Samples Kruskal–Wallis and chi square tests were used to determine statistical significance.

### Wound healing

**Figure 1:**
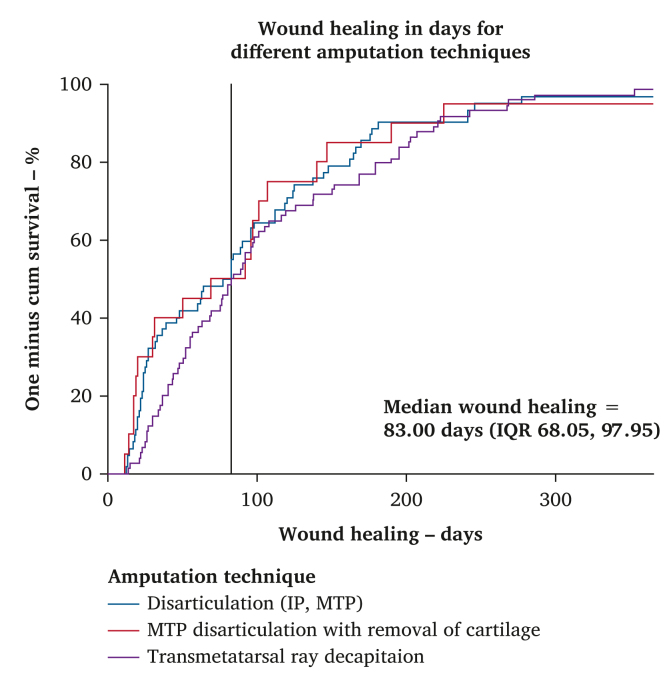
Cumulative Kaplan–Meier estimate of wound healing time by amputation technique. cum = cumulative; MTP = metatarsophalangeal.

Overall, 68.0% of patients were defined as having a healing wound at thirty days. No statistically significant difference in wound healing status for different amputation techniques was observed (MTP disarticulation 75.0%, MTP disarticulation with cartilage removal 85.7%, and transmetatarsal ray amputation 66.3%, *p* = 0.17). The median time to wound healing for the entire cohort was 83 days (IQR 31.3, 146.3 days), which was similar across amputation techniques (*p* = 0.60, Fig. 1). As expected, healing status at 30 days was significantly associated with the median time to complete wound healing: patients who had healing wounds at 30 days showed a shorter median time to complete wound healing compared with those who had non-healing wounds at 30 days (53.3 days (IQR 25.3, 97.8 days) *vs.* 168 days (IQR 101, 221 days); *p* < 0.001).

Table 2 shows the result of uni- and multivariable association with wound healing status at thirty days. Toe amputation technique was not associated with wound healing (*p* = 0.52). Presence of diabetes mellitus (odds ratio [OR] 2.41, 95% confidence interval [CI] 1.07 – 5.45; *p* = 0.034), a peri-operative toe pressure < 30 mmHg (OR 2.50, 95% CI 1.16–5.42; *p* = 0.020), an acute setting (OR 2.02, 95% CI 1.02–4.01; *p* = 0.044), a wound left open (OR 2.91, 95% CI 1.15–5.84; *p* = 0.003), and a positive per-operative culture (OR 4.05, 95% CI 0.45–34.4; *p* = 0.029) were associated with a non-healing wound 30 days after surgery. Multivariable analysis confirmed an independent association with a non-healing wound status at thirty days for diabetes and toe pressure < 30 mmHg. Given the heterogeneity of the cohort with respect to toe pressure, a sensitivity analysis restricted to patients with a toe pressure ≥ 30 mmHg was performed. This analysis yielded results consistent with the primary analysis and did not demonstrate a significant association between amputation technique and healing.

**Table 2:** Uni- and multivariable analyses for factors associated with post-operative non-healing wound status at 30 day follow up.

Risk factors	Univariable, odds ratio (95% CI)	*p* value	Multivariable, odds ratio (95% CI)	*p* value
*Smoking status*		0.88		0.85
Never smoked or ex smoker	1.00		1.00	
Active smoker	1.06 (0.48–2.37)		0.92 (0.36–2.31)	
*Diabetes*		0.034		0.021
No	1.00		1.00	
Yes	2.41 (1.07–5.45)		3.17 (1.19–8.46)	
*Setting*		0.044		0.23
Planned	1.00		1.00	
Acute	2.02 (1.02–4.01)		1.64 (0.74–3.71)	
*Peri-operative toe pressure*		0.020		0.004
≥30 mmHg	1.00		1.00	
<30 mmHg	2.50 (1.16–5.42)		4.19 (1.59–11.02)	
*Amputation technique*		0.18		0.52
MTP disarticulation	1.00		1.00	
MTP disarticulation with cartilage removal	0.50 (0.13–1.92)		0.41 (0.090-1.87)	
Transmetatarsal ray decapitation	1.53 (0.74–3.16)		0.86 (0.37–2.02)	
*Wound closure*		0.003		0.059
Transcutaneous stitches	1.00		1.00	
Left open	2.91 (1.45–5.84)		2.15 (0.97–4.76)	
*Per-operative cultures*		0.029		0.30
Negative	1.00		1.00	
Positive	4.05 (0.48–34.4)		2.28 (0.24–21.73)	
Not performed	1.60 (0.18–14.1)		1.20 (0.12–11.99)	

CI = confidence interval; MTP = metatarsophalangeal.

Because suspected infection may have been the reason for leaving the wound open, the association between wound closure and per-operative cultures was explored. Indeed, leaving the wound open was associated with taking per-operative cultures more frequently (open 69.8% *vs.* closed 51.3%; *p* = 0.018), and most cultures were positive (open 95.5% *vs.* closed 89.8%; *p* = 0.46). Moreover, because the underlying infection may explain a worse wound healing status in the acute setting, the association between setting and per-operative cultures was explored. Indeed, patients operated on in the acute setting more often had a per-operative culture taken (acute 70.2% *vs.* elective 46.8%; *p* = 0.002), and most were positive (94.9% *vs.* 88.6%; *p* = 0.28). Additionally, median peri-operative toe pressure was similar for patients in an acute *vs.* an elective setting (acute 38 mmHg *vs.* elective 36 mmHg; *p* = 0.23).

### Secondary outcomes

Table 3 shows the secondary outcomes by amputation technique. The overall incidence of local wound complications and systemic complications were 31.5% and 23.6%, respectively. No differences were observed across amputation techniques, but there were significant differences in length of stay. Patients with a transmetatarsal ray amputation had a longer median admission duration (16 days; IQR 7.0, 24.0 days) compared with those in the disarticulation group (seven days; IQR 1.0, 15.3 days) and the disarticulation with cartilage removal group (four days; IQR 1.0, 21.0 days) (*p* < 0.001).

**Table 3:** Secondary outcomes during follow up, by amputation technique.

Outcome	MTP disarticulation (*n* = 70)	MTP disarticulation with cartilage removal (*n* = 21)	Transmetatarsal ray decapitation (*n* = 87)	*p* value∗
*Wound complications within 30 d after surgery*†				0.42
Yes	20 (30.3)	5 (23.8)	31 (37.3)	
No	46 (69.7)	16 (76.2)	52 (62.7)	
*Other complications during admission*‡				0.42
Yes	13 (18.6)	5 (23.8)	24 (27.6)	
No	57 (81.4)	16 (76.2)	63 (72.4)	
Admission duration – d	7 [1.0, 15.3]	4 [1.0, 21.0]	16 [7.0, 24.0]	<0.001
*Re-admission within six mo after surgery*				0.31
Yes	16 (22.9)	3 (14.3)	15 (17.2)	
No	54 (77.1)	18 (85.7)	72 (82.8)	
*Additional revascularisations during follow up*				0.069
Yes	19 (29.2)	2 (9.5)	30 (35.3)	
No	46 (70.8)	19 (90.5)	55 (64.7)	
*Additional amputation during follow up*§				
Minor amputation	24 (36.9)	3 (14.3)	23 (26.4)	0.18
Major amputation	3 (4.3)	2 (9.5)	8 (9.2)	0.45

Data are provided as *n* (%) or median [interquartile range]. MTP = metatarsophalangeal.

∗ Pearson chi square test for categorical variables and Mann–Whitney *U* test for admission duration.

† Infection, dehiscence, or ongoing necrosis.

‡ Complications included pneumonia, heart failure, kidney injury, cardiac arrest, delirium, and electrolyte disorders.

§ Minor amputations: toe and forefoot; major amputation: transtibial, through knee, or transfemoral amputation.

### Follow up

**Figure 2:**
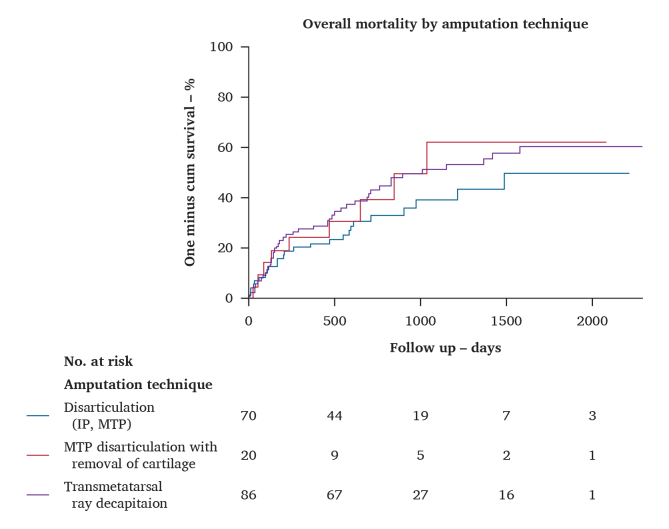
Cumulative Kaplan–Meier estimate of overall mortality by amputation technique. MTP = metatarsophalangeal.

The median follow up was 1.6 years (IQR 0.63, 3.23 years), with eight patients (3.8%) being lost to follow up. Patients had, on average, 12.5 (standard deviation 8.82) visits to the outpatient clinic within the first year after index surgery. During follow up, 51 patients (28.7%) underwent additional revascularisation procedures to the index limb to improve wound healing (Table 3). The reasons for additional revascularisations were non-healing wounds or a toe pressure of < 30 mmHg (e.g., loss of patency of previous revascularisation). In 33 of the 51 patients, revascularisation had also been performed within six months before surgery. Fifty-two revascularisation procedures in 42 patients (23.6%) were performed in the first six months following index surgery; 49 patients (27.5%) underwent 62 revascularisation procedures within the first year after index surgery. The median toe pressure of patients undergoing revascularisation within 12 months after index surgery was 36 mmHg (IQR 16.0, 71.5 mmHg). Moreover, 55 patients (30.9%) underwent an additional minor (*n* = 50, 28.1%) or major (*n* = 13, 7.3%) amputation, which was not associated with amputation technique (*p* = 0.55). These additional amputations were performed owing to non-healing wounds, progression of infection, or new (transfer) ulcers. Among these, 16 patients had a recorded toe pressure of < 30 mmHg at index surgery. Forty-six (25.8%) patients underwent an additional amputation in the first six months after index surgery. In the first year after index surgery, 51 patients (28.7%) underwent an additional amputation. Seventy-eight patients (43.8%) died during follow up, including 35 patients (19.7%) who died within the first six months after index surgery. As depicted in Figure 2, amputation technique was not associated with overall mortality (MTP disarticulation as reference; MTP disarticulation with cartilage removal hazard ratio 1.39, 95% CI 0.65–2.99; transmetatarsal ray decapitation hazard ratio 1.41, 95% CI 0.86–2.30; *p* = 0.37).

## Discussion

The primary objective of this cohort study was to evaluate the association between different toe amputation techniques and wound healing outcomes in patients with CLTI. Amputation technique was not associated with wound healing. Importantly, vascular status appeared to be the dominant determinant of wound healing outcomes in this cohort because a toe pressure or TcPO_2_ < 30 mmHg was independently associated with impaired healing. This finding underscores that ischaemic burden may outweigh technical aspects of toe amputation when evaluating post-operative outcomes in patients with CLTI.

To the authors’ knowledge, this is the first study comparing three toe amputation techniques in patients with CLTI. An observational study by Pirozzi *et al.*^23^ showed similar wound healing time after amputation, irrespective of removing or maintaining the cartilage cap. However, only 63 patients were included, and the inclusion criteria were infection and trauma, rather than PAD. A systematic review by Aerden *et al.*^14^ showed that cartilage removal was not associated with wound healing and infection after amputations. However, inclusion was restricted to Chopart, ankle, and knee disarticulations.

Although the majority of patients had a healing wound status at 30 days, the time to complete wound healing was long, underscoring the vulnerable status of these wounds and patients. Moreover, the mean time to wound healing in those with non-healing status at thirty days was even longer, which might be an indication of poor vascular status, e.g., that there were no revascularisation options. However, there might also be insufficient awareness of non-healing status at 30 days, resulting in a delay in additional revascularisation. Prolonged time to wound healing probably reflects advanced ischaemic disease in a subset of patients, particularly those with limited or no remaining options for revascularisation. In these cases, persistent non-healing may represent the natural course of end stage CLTI rather than failure of the index amputation. Post-operative perfusion measurements were not routinely repeated once revascularisation options were exhausted because, reflecting real world practice, results were unlikely to alter clinical management.

Secondary outcomes were not associated with amputation technique. Overall, the incidence of complications, additional minor and major amputations, and further revascularisation procedures was high. Notably, a high proportion of patients were operated on in an acute setting. This may not only reflect the unpredictable and sometimes fast deterioration of disease but also be an indication of underestimated pre-operative assessment. A substantial proportion of patients had undergone revascularisation within six months before toe amputation, and some required additional revascularisation during follow up. This highlights the progressive and recurrent nature of CLTI, in which repeated vascular interventions and amputations are often part of the disease trajectory rather than isolated treatment failures.

Several patient and disease related factors were associated with poor wound healing in this study. In addition to impaired pedal perfusion, diabetes mellitus, positive per-operative cultures, and acute presentation were more frequently observed in patients with delayed or absent healing. These findings are consistent with the existing literature and reinforce that systemic comorbidity and disease severity substantially influence outcomes after minor amputation for CLTI. Although smoking has been identified as a predictor of impaired wound healing in previous studies, this association was not observed in the analysis.^6^, ^7^, ^8^, ^9^, ^10^, ^11^, ^12^

The current study has several strengths and limitations. The larger sample size and follow up duration of a real life population are a significant strength when compared with other studies on toe amputation. Additionally, there is little loss to follow up. Being a retrospective cohort study, amputation technique is not randomised. Clinical status, as well as surgeon preference and experience, may affect the choice of treatment, thereby inducing confounding by indication. The heterogeneity in vascular status, including patients with no-option disease, previous revascularisation, and peri-operative revascularisation, limits the ability to isolate the effect of amputation technique. Moreover, retrospective assessment of wound healing status is less reliable. However, most patients undergo a standardised wound healing assessment at 30 days (±5 days) after surgery as part of routine clinical follow up for amputation surgery at this centre. Of note, no deviations from this timeframe were observed in patients who survived until post-operative day 25. Post-operative offloading strategies and participation in rehabilitation programmes were not included in this analysis. Furthermore, complete data on time from origin of the wound to revascularisation, infection, post-operative toe pressure measurements, and affected arterial territory were not available*.* These factors probably influence healing outcomes and functional recovery and should be addressed in future research to provide a more comprehensive understanding of post-operative trajectories in this patient population. This limitation reflects the retrospective design and real world nature of the cohort.

Several lessons can be learned from the current study. Firstly, the results did not favour a certain amputation technique. However, firm conclusions regarding amputation technique are to be drawn by a randomised study, or pseudo randomisation using an instrument variable. Secondly, even in those with a positive healing status at 30 days, time to wound healing is long, despite all efforts to optimise vascular status, treat infection, and ensure dedicated wound care. This warrants more emphasis on peri-operative protocols, including pro-active surgical planning, standardised peri-operative antibiotic regimens and culture strategies, and aggressive re-interventions for a non-healing wound at 30 days. Early identification of non-healing wounds at 30 days should prompt timely reconsideration of revascularisation or surgical strategy. Thirdly, the high morbidity and mortality rates reflect the vulnerability of this population and the need to incorporate advanced care planning to carefully balance different treatment options. Conservative or palliative care may be a reasonable alternative for those most vulnerable. Moreover, a more radical amputation as a primary intervention might improve wound healing and quality of remaining life. Future research may focus on prediction of which patients might benefit most from this approach to accommodate the life and treatment goals of these frail patients.

### Conclusions

Despite the relatively limited extent of these amputations, wound healing was prolonged and rates of re-intervention, subsequent amputation, and death were high, reflecting the severe systemic and vascular disease burden in this population. Diabetes mellitus and impaired pedal perfusion were independently associated with early non-healing, underscoring the importance of meticulous pre-operative vascular assessment and optimisation. These findings suggest that the choice of toe amputation technique should primarily be guided by local wound characteristics, infection control, and surgeon judgement rather than expectations of superior healing outcomes. Given the substantial observed morbidity and mortality rates, careful patient selection, timely reassessment of wound healing, and consideration of alternative treatment strategies, including more proximal amputation or palliative approaches in selected patients, are essential to optimise care in this vulnerable population.

## Funding

This research did not receive any specific grant from funding agencies in the public, commercial, or not for profit sectors.

## Declaration of Generative AI and AI-assisted Technologies in the Writing Process

During the preparation of this work the authors used ChatGPT to enhance the readability of this article. After using this tool/service, the authors reviewed and edited the content as needed and take full responsibility for the content of the publication.

## Conflict of Interest

The authors have no competing interests.
