# Supplementary material for: Operation Techniques and Outcomes in Patients with Chronic Limb Threatening Ischaemia Undergoing Minor Amputations—A Cohort Study
Source: EJVES Vasc Forum. 2026 May 14;66:9–16. doi: 10.1016/j.ejvsvf.2026.05.001 (PMC13273673; doi:10.1016/j.ejvsvf.2026.05.001)
Supplement: Supplementary file 1 [file mmc1.pdf]

Supplementary Table S1. Criteria for primary amputation wound healing assessment on post-operative day 30 (± 5 days).[20]

**Category**	**Criteria**
Non-Healing	Development of necrosis<br>Development of infection, including gangrene or abscess<br>Ulceration occurring within or adjacent to the surgical wound<br>Disruption or dehiscence of suture line<br>Drainage or exudate expressed from suture line<br>Evidence of inflammatory response including swelling, cellulitis, or skin discoloration<br>Hematoma formation<br>Revision of the amputation to a more proximal level
Healing	Re-epithelialization of tissue within the incision site<br>Absence of all criteria for non-healing wounds

REFERENCES

20. Squiers JJ, Thatcher JE, Bastawros DS, Applewhite AJ, Baxter RD, Yi F, et al. Machine learning analysis of multispectral imaging and clinical risk factors to predict amputation wound healing. *J Vasc Surg* 2022;**75**:279–85.
